# YAP regulates PD-L1 expression in human NSCLC cells

**DOI:** 10.18632/oncotarget.23051

**Published:** 2017-12-09

**Authors:** Jinbai Miao, Ping-Chih Hsu, Yi-Lin Yang, Zhidong Xu, Yuyuan Dai, Yucheng Wang, Geraldine Chan, Zhen Huang, Bin Hu, Hui Li, David M. Jablons, Liang You

**Affiliations:** ^1^ Thoracic Oncology Laboratory, Department of Surgery, Comprehensive Cancer Center, University of California, San Francisco, CA, USA; ^2^ Department of Thoracic Surgery, Beijing Chao-Yang Hospital, Affiliated with Capital Medical University, Beijing, People’s Republic of China; ^3^ Department of Thoracic Medicine, Chang Gung Memorial Hospital, Linkou, Taoyuan, Taiwan; ^4^ Department of Hepatobiliary Surgery, National Cancer Center/Cancer Hospital, Chinese Academy of Medical Sciences and Peking Union Medical College, Beijing, China; ^5^ Class of 2020, Medical College of Wisconsin, Milwaukee, WI, USA

**Keywords:** programmed death-ligand 1, yes-associated protein, non-small cell lung cancer, hippo pathway

## Abstract

Programmed death-ligand 1 (PD-L1) is a membrane protein on tumor cells that binds to the PD-1 receptor expressed on immune cells, leading to the immune escape of tumor cells. Yes-associated protein (YAP) is a main effector of the Hippo/YAP signaling pathway, which plays important roles in cancer development. Here we show that YAP regulates PD-L1 expression in human non-small cell lung cancer (NSCLC) cells. First, we investigated YAP and PD-L1 expression at the protein level in 142 NSCLC samples and 15 normal lung samples. In tumor tissue, immunohistochemistry showed positive staining for YAP and PD-L1, which correlated significantly (*n* = 142, *r* = 0.514, *P* < 0.001). Second, in cell lines that express high levels of PD-L1 (H460, SKLU-1, and H1299), the ratio of p-YAP/YAP was lower and GTIIC reporter activity of the Hippo pathway was higher than those in three cell lines expressing low levels of PD-L1 (A549, H2030, and PC9) (*P* < 0.05). Third, in the same three cell lines, inhibition of YAP by two small interfering RNAs (siRNAs) decreased the mRNA and protein level of PD-L1 (*P* < 0.05). Fourth, forced overexpression of the YAP gene rescued the PD-L1 mRNA and protein level after siRNA knockdown targeting 3′UTR of the endogenous YAP gene. Finally, chromatin immunoprecipitation (ChIP) assays using a YAP-specific monoclonal antibody resulted in the precipitation of PD-L1 enhancer region encompassing two putative TEAD binding sites. Our results indicate that YAP regulates the transcription of PD-L1 in NSCLC.

## INTRODUCTION

Lung cancer remains the leading cause of cancer-related mortality worldwide [1, 2], and non-small cell lung cancer (NSCLC) accounts for 80% of cases. Despite some improvements in treatment, the overall prognosis for NSCLC remains poor. The most common treatment plan for NSCLC is surgical resection combined with chemotherapy [3]. Platinum-based chemotherapy is the standard first-line treatment for advanced disease, but its role is limited due to its side effects. NSCLC patients acquire resistance to multiple therapeutic modalities, leading to a 5-year survival rate of approximately 16% [4]. Targeted therapy has shown a better clinical effect for advanced NSCLC, but is currently only available for patients whose tumors have mutations such as epidermal growth factor receptor (EGFR) or anaplastic lymphoma kinase (ALK). The acquired resistance to targeted therapy also limits its ability to prolong survival [5]. Novel therapeutic strategies, including immunotherapy, are under investigation, but more useful biomarkers and therapeutic targets for patients with advanced NSCLC are needed.

PD-L1 (also known as B7-H1 or CD274), a type I transmembrane surface glycoprotein encoded by the CD274 gene, promotes T-cell tolerance and escapes host immunity by inhibiting CD8+ T-cell survival, effector function, and inducing Fas-mediated T-cell apoptosis [6]. PD-L1 is expressed on the surface of tumor cells, including in NSCLC, melanoma, and breast cancer. PD-L1 expression is significantly correlated with tumor related genes such as KRAS, p53, EGFR, and others [7, 8]. In the tumor microenvironment, PD-L1 expression is greatly upregulated by LPS and interferon-γ (IFN-γ) in a STAT1/3-dependent manner [9]. Although immune checkpoint inhibitors targeting the PD-1/PD-L1 pathway in NSCLC have shown promising results, with ∼30% of tumors responding [10–13], resistance is common. A better understanding of the regulatory mechanism of PD-L1 may help identify biomarkers and/or develop combinatorial strategies for clinical use.

Overexpression of the transcription coactivator Yes-associated protein (YAP) has been found in many cancers due to abnormal amplification, loss of Hippo signaling by mutation, and/or downregulation of the core Hippo component [14]. YAP overexpression also contributes to self-renewal, tumor initiation capacity and resistance to anticancer drugs [15–18]. The role of YAP in tumor immunity has just begun to be explored [19]. The study by Moroishi *et al*. [14] indicates that hyperactivation of YAP and TAZ significantly contributes to tumor growth suppression through TEAD-mediated transcription. Another study found that YAP is a negative regulator of innate immunity through interaction with IRF3 [20]. Although these limited studies have demonstrated that Yap plays a role in tumor immunity, the effects of YAP on tumor growth, especially in the context of reciprocal interactions between tumor cells and host anti-tumor immune responses, remain largely unknown because of the complexity of tumorigenesis and immune regulation.

In this study, we hypothesized that YAP is involved in the regulation of PD-L1 expression in NSCLC. To test this, we evaluated YAP and PD-L1 expression in human NSCLC tissues and then investigated whether PD-L1 is a downstream target of YAP in NSCLC cell lines.

## RESULTS

### YAP and PD-L1 are co-expressed in NSCLC tissues

**Figure 1:**
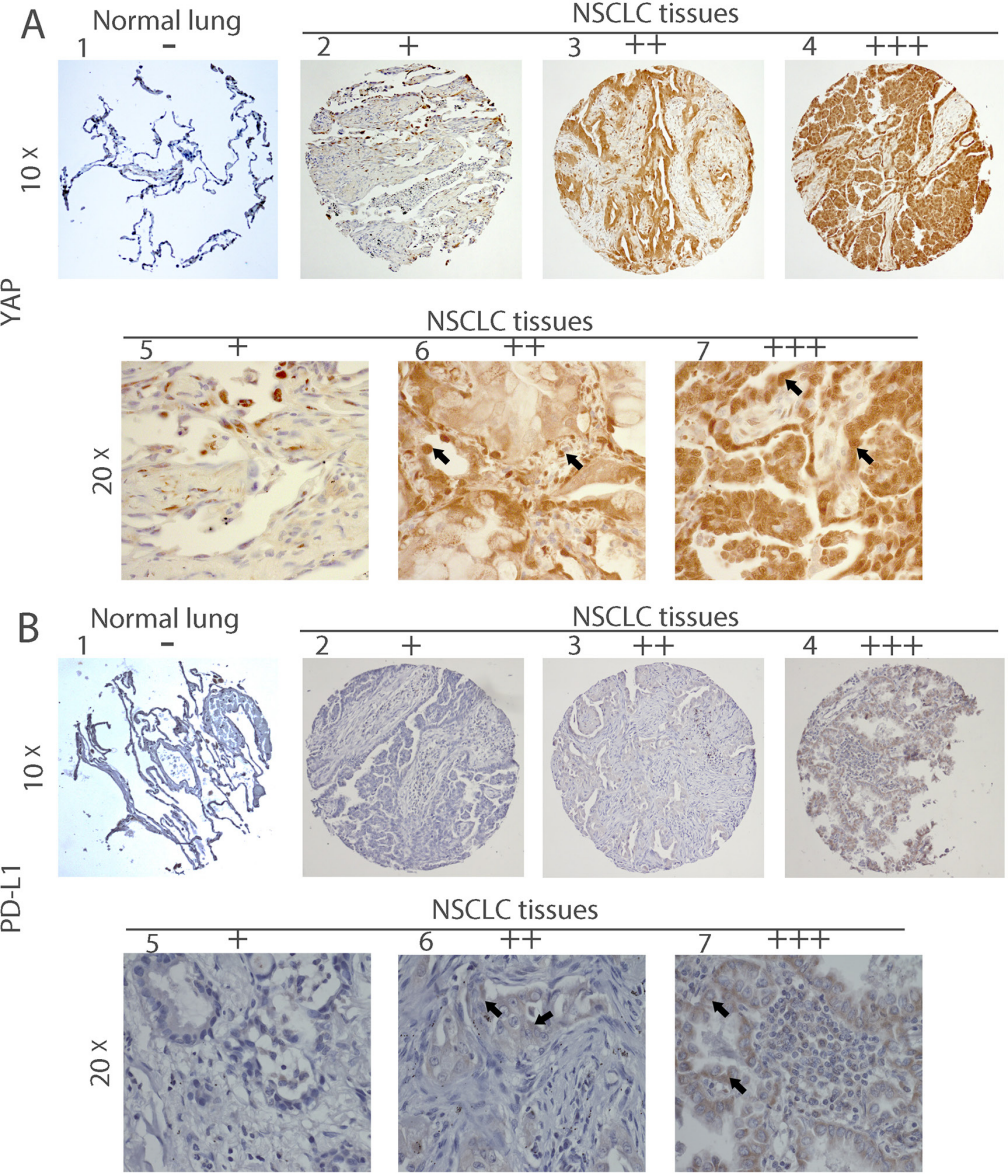
Immunohistochemistry of YAP and PD-L1 in human NSCLC tissues Representative image showing expression of YAP protein (**A**) and PD-L1 protein (**B**) in human NSCLC tissues and normal lung tissues analyzed by immunohistochemistry. (**A:1**) and (**B:1**) are normal lung tissues. (**A:2–7**) and (**B: 2–7**) are NSCLC tissues. (**A:5–7**) Staining of YAP was localized in nuclei (arrow) and **(B:5–7)** staining of PD-L1 was localized in membrane (arrow), under a 20× objective lens. – and + mean negative; ++ and +++ mean positive.

YAP is an important mediator protein in cancer development. Programmed death-ligand 1 (PD-L1; also called B7-H1 or CD274), which is expressed on many cancer cells, is one of the most important inhibitory molecules that promote tumor immune escape. To investigate the relationship of YAP and PD-L1 in NSCLC tissues, we used immunohistochemistry (IHC) to examine their protein level in 142 samples of primary human NSCLC and 15 normal lung samples (Figure 1). We found that 88 cases (62.0%) were YAP-positive and 52 cases (36.6%) were PD-L1 positive (Table 1, Supplementary Table 1). Forty-three cases (30.3%) were both YAP and PD-L1 positive, accounting for, respectively, 48.9% of YAP-positive samples and 82.7% of PD-L1 positive samples. PD-L1 positive samples showed significantly higher YAP positive ratios compared to PD-L1 negative samples (X^s^ = 14.947, *P* < 0.001). In normal lung tissues, no cases were positive for YAP or PD-L1 (Table 2). There was no significant difference in YAP and PD-L between different pathological types and TNM stage (*P* > 0.05) (Supplementary Tables 2, 3). To further analyze the relationship between YAP and PD-L1, we performed a Spearman product correlation test. YAP and PD-L1 were mildly, but still significantly correlated at the protein level (*n* = 142, r = 0.514, *P* < 0.001).

**Table 1:** YAP and PD-L1 IHC comparison in 142 human primary NSCLC tissues

YAP						
		Positive (++/+++)	Negative (−/+)	Total	X^2^	*P* value
PD-L1	Positive (++/+++)	43	9	52		
	Negative −/+)	45	45	90		
Total		88	54	142	14.947	<0.001

− = no stain; + = weak stain; ++ = moderate stain; +++ = strong stain; ++/+++ = positive; −/+ = negative.

**Table 2:** YAP and PD-L1 IHC comparison in normal human lung tissues

		YAP		
		Positive (++/+++)	Negative (−/+)	Total
PD-L1	Positive (++/+++)	0	0	0
	Negative (−/+)	0	15	15
Total		0	0	

− = no stain; + = weak stain; ++ = moderate stain; +++ = strong stain; ++/+++ = positive; −/+ = negative.

### YAP and PD-L1 are co-expressed in NSCLC cell lines

**Figure 2:**
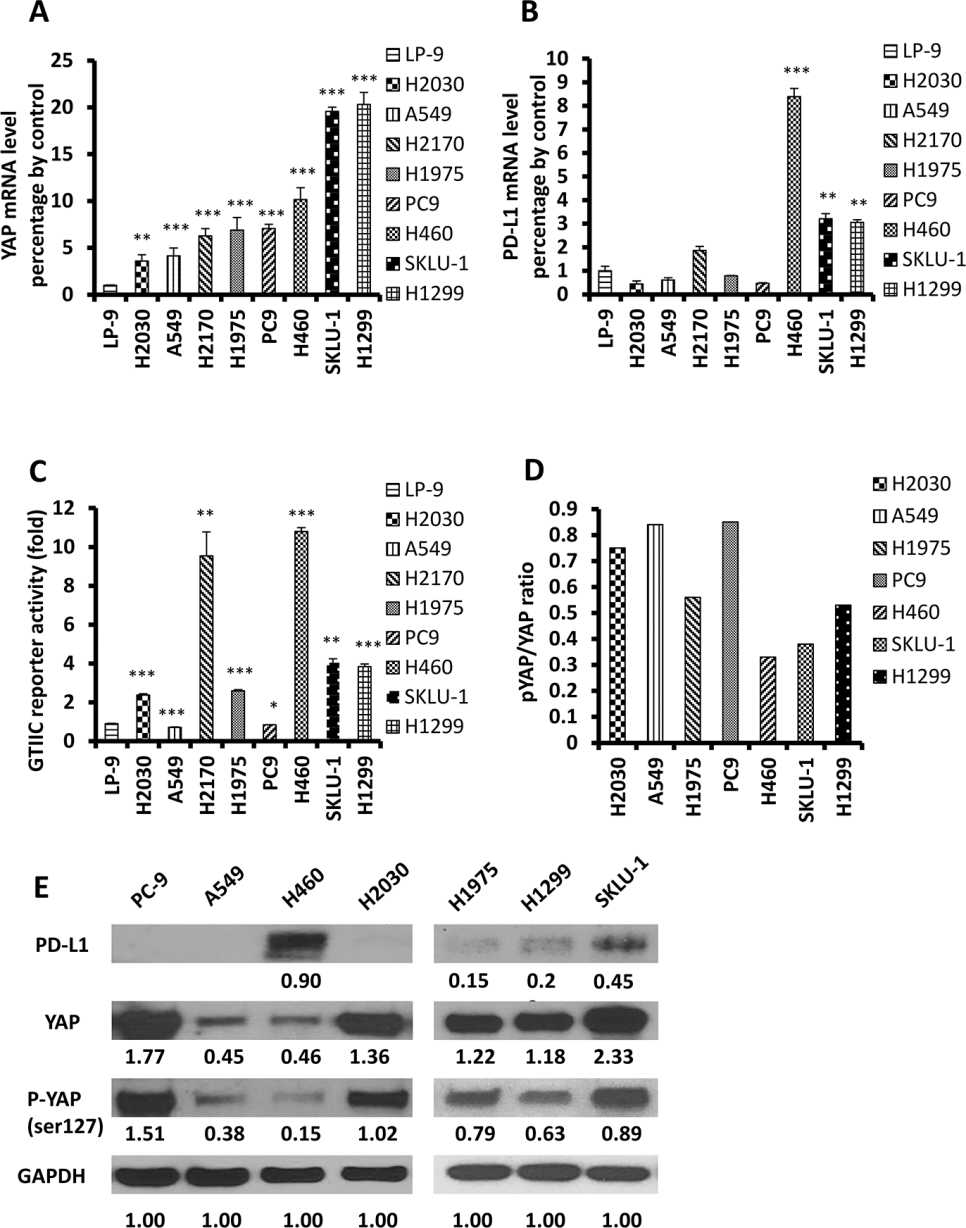
Expression of YAP and PD-L1 in NSCLC cell lines (**A**–**B**) The mRNA levels of YAP and PD-L1 in NSCLC cell lines were measured using qRT-PCR, and LP-9 cell line was used as control (F = 174.10 *P* < 0.001; F = 635.77 *P* < 0.001). VS LP-9: ^*^P < 0.05, ^**^P ≤ 0.01, ^***^P ≤ 0.001. (**C**): GTIIC reporter activity of the Hippo pathway in NSCLC cell lines, and LP-9 cell line was used as control (F = 311.39; *P* < 0.001). VS LP-9: ^*^P < 0.05, ^**^P ≤ 0.01, ^***^P ≤ 0.001. (**D**) pYAP/YAP ratio in NSCLC cell lines based on the value of Western blot. (**E**) Western blot was used to detect levels of YAP, pYAP and PD-L1 in NSCLC cell lines. GAPDH was detected as a loading control. Band intensity was analyzed with ImageJ software and normalized with the intensity of GAPDH band.

To elucidate the relationship between YAP and PD-L1, we further studied eight NSCLC cell lines: H460, H2170, SKLU-1, H1975, H1299, A549, H2030 and PC9. We analyzed GTIIC reporter activity of Hippo pathway and found higher activity in H460, H2170, SKLU-1, H1299, H1975, and H2030 cell lines than in A549, PC9 and LP-9 (*P* < 0.05; Figure 2C, Supplementary Table 6). We then used qRT-PCR to detect mRNA expression. In SKLU-1 and H1299 cell lines, the PD-L1 and YAP mRNA levels were significantly higher than in the other cell lines (*P* < 0.05; Figure 2B, Supplementary Table 5). In H460 cells, PD-L1 mRNA expression was the highest (*P* < 0.001), but YAP mRNA expression was lower than that in SKLU-1 and H1299 cell lines (*P* < 0.001), higher than that in A549, H2170 and H2030 cell lines (*P* < 0.01), and the same as that in H1975 and PC9 cell lines (Figure 2A, Supplementary Table 4). Next, we used western blot to detect protein expression, and found that the p-YAP (ser127) /YAP ratio decreased significantly in cell lines that expressed high levels of PD-L1 (H460, SKLU-1, and H1299) (Figure 2D and 2E). YAP was stained in both nucleus and cytoplasm, whereas pYAP (ser127) was found in the cytoplasm (Supplementary Figure 1A, 1B, 1E). Then we detected protein expression of pYAP (Tyr357), src, and TAZ in H2030, PC9 and A549 cells with different degrees of YAP and pYAP (ser127) expression. The protein expression level of p-YAP (ser127) was higher than that of pYAP (Tyr357) in H2030 and PC9 cell lines. In A549 cell lines, the expression of pYAP (Tyr357) was higher than in H2030 and PC9 cell lines. (Supplementary Figure 1C). These results suggest that YAP and PD-L1 are co-expressed in H460, SKLU-1, and H1299 cell lines.

### Inhibition of YAP downregulates PD-L1 expression in H460, SKLU-1, and H1299 cell lines

**Figure 3:**
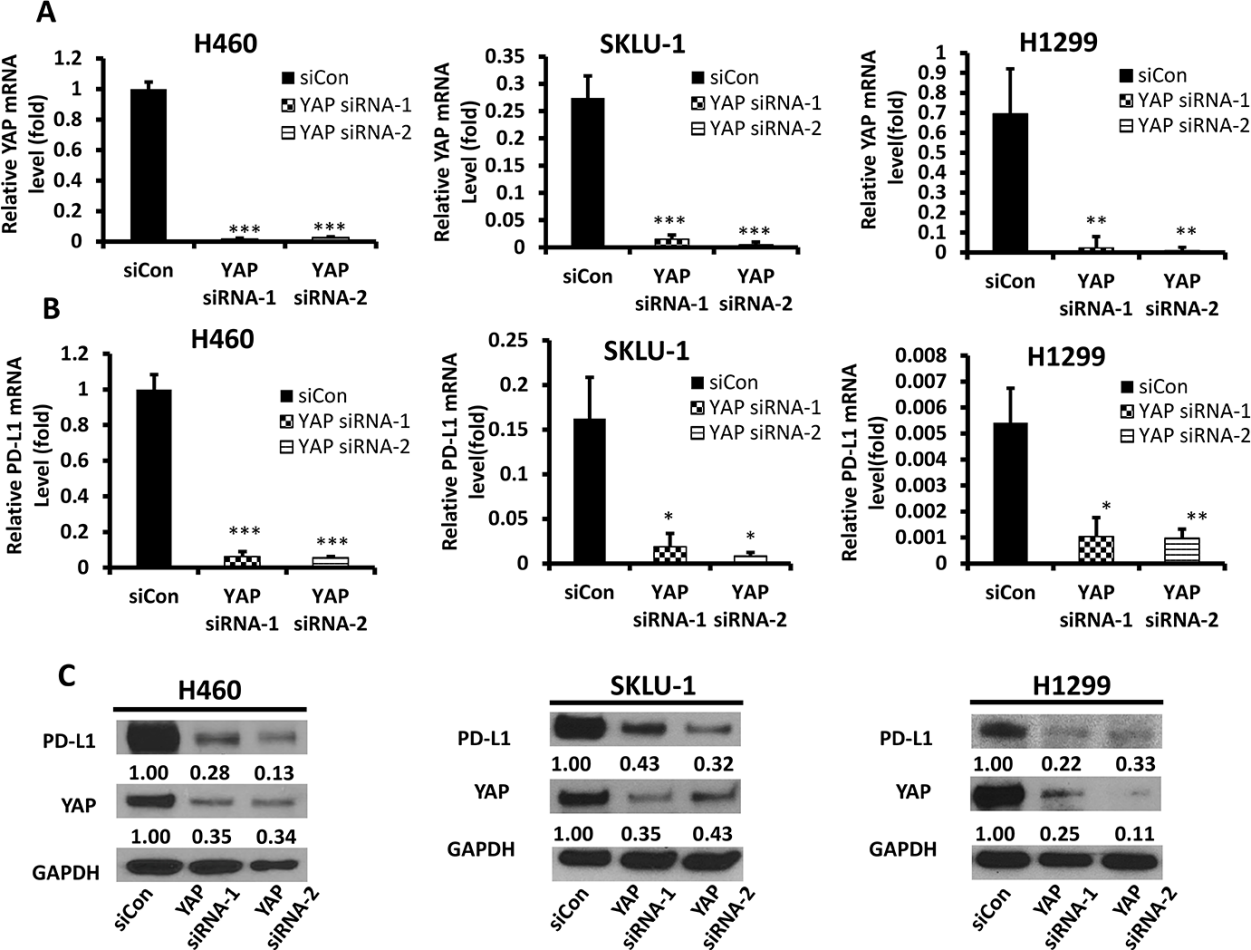
YAP and PD-L1 expression in H460, SKLU-1 and H1299 cell lines after knockdown of YAP (**A**) YAP mRNA level in H460, SKLU-1 and H1299 cells after YAP inhibition by siRNA1,2 was measured using qRT-PCR. (**B**) PD-L1 mRNA level in H460, SKLU-1 and H1299 cells after YAP inhibition by siRNA1,2 was measured using qRT-PCR. (**C)** Western Blot analysis in H460, SKLU-1 and H1299 cell lines transfected with YAP siRNA1,2 or control siRNA. GAPDH was detected as a loading control. Band intensity was analyzed with ImageJ software and normalized with the intensity of GAPDH band. YAP-knockdown reduced PD-L1 expression significantly. ^*^P < 0.05, ^**^P ≤ 0.01, ^***^P ≤ 0.001.

To further understand whether YAP can regulate PD-L1, siRNA-YAP (3′ and 5′) was used to silence the YAP gene in H460, SKLU-1 and H1299 cell lines. The mRNA and protein levels of PD-L1 were detected, respectively, by qRT-PCR and Western blot. The results confirmed that the YAP gene was significantly inhibited by transfection of siRNA-YAP (Figure 3A). qRT-PCR showed a significant decrease in PD-L1 mRNA levels in the H460, SKLU-1 and H1299 cell lines after 48 hours of treatment with siRNA-YAP (3′ and 5′UTR) (*P* < 0.05) (Figure 3B). Western blot confirmed that the protein expression was also reduced (Figure 3C). After YAP gene silencing, the protein expression of YAP and pYAP (ser127) were significantly decreased, but the protein expression levels of Src, TAZ, and pYAP (Tyr357) were no significantly changed (Supplementary Figure 1D). Together, these results suggest that PD-L1 expression is regulated by YAP expression in NSCLC cells.

### Forced overexpression of the YAP gene rescued PD-L1 mRNA and protein levels

**Figure 4:**
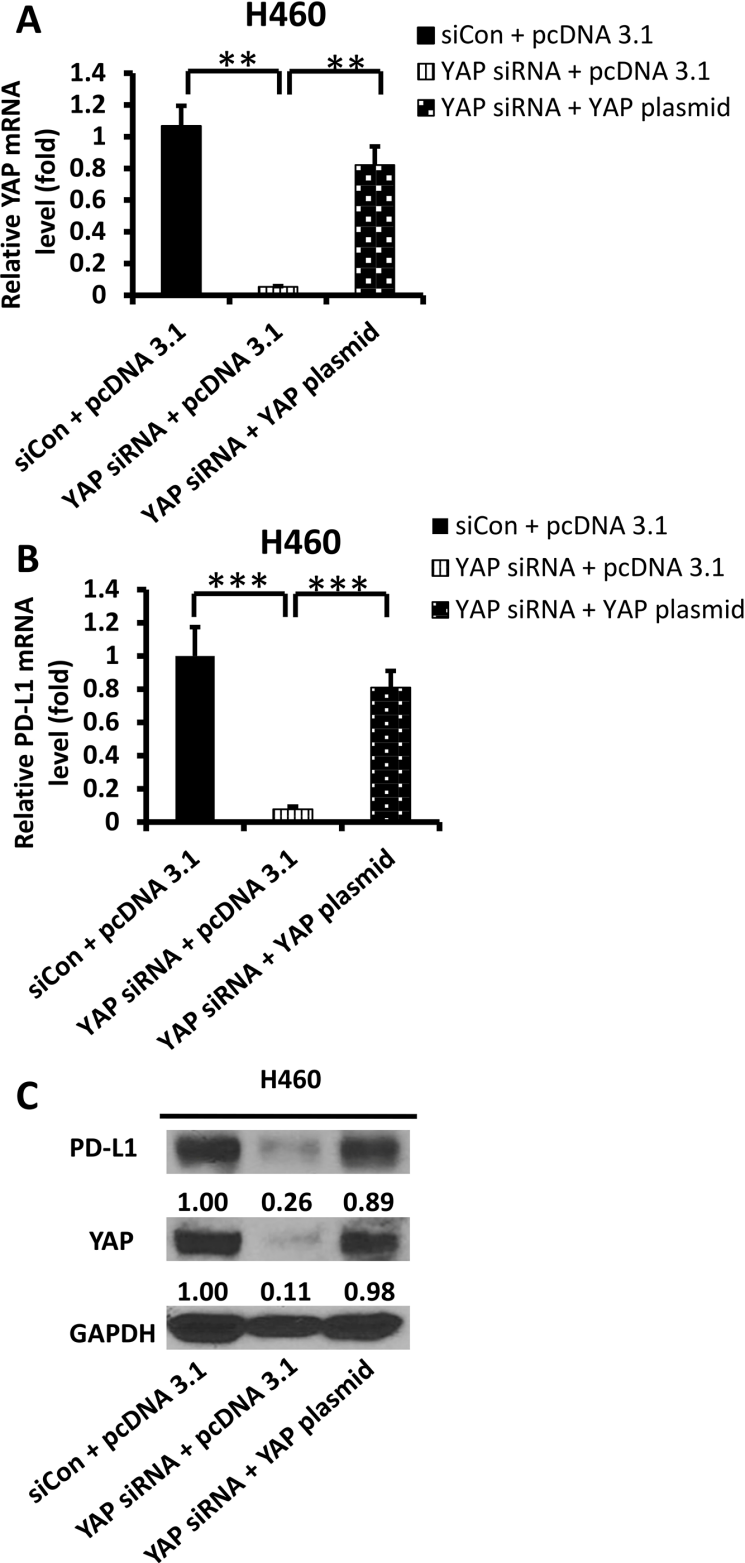
Expression of PD-L1 and YAP after YAP forced over-expression in siRNA-YAP H460 cells (**A**–**B**) qRT-PCR analysis of mRNA levels of YAP and PD-L1 after YAP silencing by siRNA and/or forced over-expression of the YAP gene in H460 cells. (**C**) Western blot analysis of YAP, PD-L1 after YAP silencing by siRNA and/or forced over-expression of the YAP gene in H460 cells. GAPDH was detected as a loading control. Band intensity was analyzed with ImageJ software and normalized with the intensity of GAPDH band. ^*^P < 0.05, ^**^P ≤ 0.01, ^***^P ≤ 0.001.

To confirm that YAP regulates PD-L1 transcription and translation, we analyzed PD-L1 mRNA and protein levels after YAP inhibition and/or forced overexpression of the YAP gene in H460 cells. We used YAP siRNA, which targeted the 3′UTR end of the YAP gene. After YAP depletion, the PD-L1 mRNA and protein levels were decreased, similar to what occurred after we inhibited YAP by using pooled YAP siRNA. To verify that the decreased PD-L1 expression was regulated by YAP knockdown, we examined PD-L1 expression level after forced YAP overexpression in siRNA-YAP H460 cell line. Using qRT-PCR and western blot analyses, we confirmed the increase in YAP mRNA and protein levels after forced YAP overexpression (*P* < 0.01) (Figure 4). These findings confirm that the YAP gene regulates PD-L1 mRNA and protein expression.

### YAP regulates PD-L1 at the transcriptional level through binding to the PD-L1 enhancer

**Figure 5:**
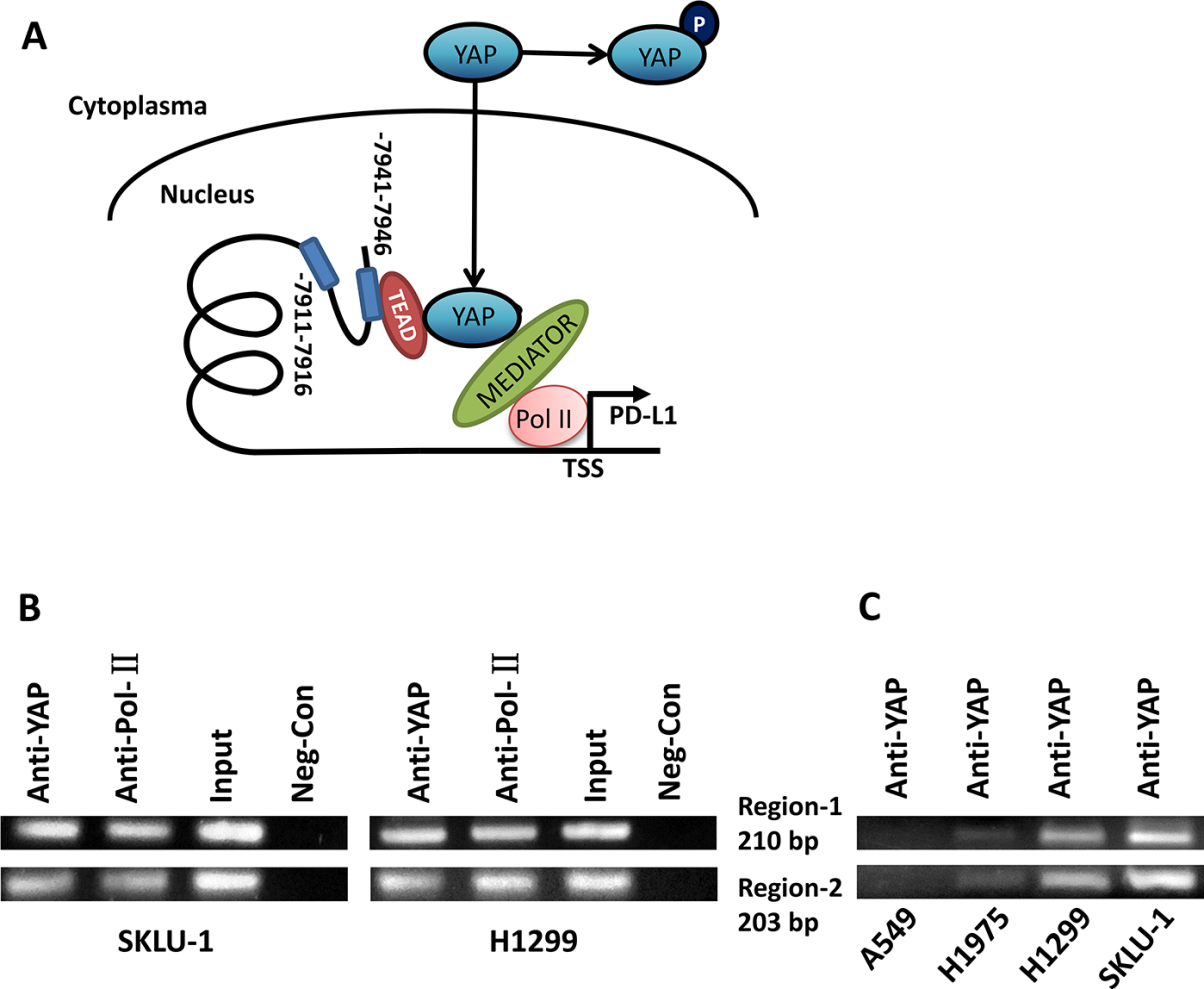
YAP regulates PD-L1 at the transcriptional level through binding to PD-L1 enhancer (**A**) Schematic of the PD-L1 promoter region. Sequence analysis revealed a putative YAP1-TEAD co-binding site between −7911 to −7941 nucleotides upstream of the transcription start site. (**B**) ChIP assays were performed with SKLU-1 and H1299 cells in two regions (203 bp and 210 bp), which were shown by gel bands of RT-PCR products with 36 cycles. (**C**) ChIP assays were performed with A549, H1975, H1299 and SKLU-1 cells in two regions (203 bp and 210 bp), which were shown by gel bands of RT-PCR products with 30 cycles.

YAP as a transcription co-activator, together with TEAD family proteins, regulates many genes, including BIRC5, CTGF and CYR61 [21]. Therefore, we asked whether YAP regulates PD-L1 at the transcriptional level. To answer this question, we examined the PD-L1 enhancer region (−10000 bps) upstream of the transcription starting site of PD-L1 and found two putative TEAD-binding sites (CATTCC), which are 7941 bps and 7911 bps upstream of the PD-L1 transcription start site (Figure 5A). We used chromatin immunoprecipitation (ChIP) to test our hypothesis in H460, SKLU-1 and H1299 cells. ChIP studies using a YAP specific monoclonal antibody resulted in the precipitation of two PD-L1 enhancer regions encompassing the putative TEAD binding site (Figure 5B). In the control ChIP assays using Rabbit IgG or without any antibody, we did not detect PD-L1 enhancer-region binding. Then we investigated the transcriptional activity change after YAP binding to PD-L1 in A549, H1975, H1299 and SKLU-1 cell lines, which express different levels of PD-L1. The results indicate that transcriptional activity differed significantly among cell lines, which was consistent with the results of Western blot expression (Figure 5C). These findings confirmed that YAP directly occupied the enhancer regions of PD-L1.

## DISCUSSION

The results of our study provide several lines of evidence to support that YAP is involved in the regulation of PD-L1 expression. First, IHC staining indicated that YAP and PD-Ll expression were significantly correlated in NSCLC tissue. Second, GTIIC reporter activity of the Hippo pathway was higher and the ratio of P-YAP/YAP was lower in cell lines that expressed high levels of PD-L1 (H460, SKLU-1, and H1299) than in other cell lines. Moreover, in H460, SKLU-1 and H1299 cells, inhibition of the endogenous YAP by siRNAs downregulated PD-L1 mRNA and protein levels, and this could be rescued by forced overexpression of YAP. Third, our ChIP results demonstrated that precipitation of the PD-L1 enhancer region encompasses the putative TEAD binding site in H460, SKLU-1 and H1299 cell lines.

In our study, YAP and PD-L1 were expressed to different degrees in NSCLC tissues. YAP, a candidate oncogene in a variety of cancers, is significantly elevated in hepatocellular cancer, prostate cancer, and breast carcinoma [22, 23]. The positive rate of YAP was 62.0% in our study, which is similar to reported rates of 54% [23] and 66.3% [24]. The PD-L1 ligand of programmed cell death 1 (PD-1) is upregulated in various types of cancers [25]. In recent clinical series, PD-L1 expression in NSCLC ranges from 7.4% to 72.7% [22, 26], possibly because of clinicopathologic characteristics and/or molecular alterations. In an analysis of 297 NSCLC cases, 43.1% of NSCLC patients had PD-L1 positive staining [27]. In our study, the positive rate of PD-L1 was 36.6% (52/142), and TNM stage and histopathology did not significantly differ between PD-L1 positive and PD-L1 negative patients (*P* > 0.05). In tumor development, YAP is thought to be associated with a variety of factors. A significant correlation between YAP and Jag-1 staining was observed in pancreatic ductal adenocarcinoma (*r* = 0.442, *p* < 0.001) [28]. However, YAP and PD-L1 co-expression in NSCLC has not been reported previously. Our study showed this co-expression in 30.3% (43/142) of NSCLC samples and that the YAP-positive ratio was higher in PD-L1 positive samples than in PD-L1 negative samples (*P* < 0.001). Expression of the two proteins was mildly, but still significantly correlated (*n* = 142, *r* = 0.514, *P* < 0.001).

We investigated eight NSCLC cell lines and found that three (H460, SKLU-1 and H1299) had higher PD-L1 expression in mRNA and protein level compared to others. Chen *et al*. [29] reported different expression levels of PD-L1 in PC9 and H1975 cell lines. In our study, the degree of PD-L1 expression was lower in H1975 than in H460, SKLU, and H1299, which was similiar to Chen *et al*’s western blot results. In their study, PD-L1 expression in H1975 cells was also stronger than in PC9 cells. We also found that high expression of PD-L1 is positively correlated with an increase of intranuclear YAP. YAP activation plays an important role in transcriptional regulation. Phosphorylation of YAP by LATS1 leads to cytoplasmic retention of YAP, which inhibits its function as a nuclear transcription coactivator [30–32]. In cell lines that express high levels of PD-L1, we found that the ratio of p-YAP/YAP was lower and GTIIC reporter activity of the Hippo pathway was higher than in cell lines that express low levels of PD-L1. The expression of pYAP (Tyr357) was weak in the cell lines we studied, which suggested that its effect may be limited in the cell lines we studied. Furthermore, silencing the YAP gene with siRNA-YAP (3′ and 5′) significantly decreased PD-L1 transcriptional level. PD-L1 expression levels were also significantly rescued after forced YAP overexpression in siRNA-YAP H460 cells. These findings suggest that YAP is involved in the positive regulation of PD-L1 expression in NSCLC cells.

YAP translocation into the nucleus, as a transcriptional coactivator, plays an oncogenic function mainly by interacting with the TEAD family of transcription factors [33, 34]. Accumulating evidence [35–38] suggests YAP expression promotes cell transformation, epithelial-to-mesenchymal transition (EMT), and cell invasion. Our previous studies have shown that YAP promotes drug resistance in NSCLC through an autocrine loop with the ERBB3 pathway, regulation of ABCG2, and side-population cell formation [15, 39]. However, the role of YAP in tumor immunity is poorly understood. A recent study [40] reported that YAP regulates the transcription of PD-L1 in EGFR-TKI-resistant PC9 cells through binding of the YAP/TEAD complex to the PD-L1 promoter, but it is unclear whether this mechanism exists in other NSCLC cell lines. The heterogeneity of PD-L1 expression levels and the promising approaches for targeted immunotherapy lung cancer treatment make it important to understand the signaling regulation of PD-L1 expression in NSCLC cells [41]. Here our results indicate that at the transcription level, YAP regulates PD-L1 expression in NSCLC cell lines. Our ChIP data performed with H460, SKLU-1 and H1299 cells demonstrate that YAP binds to the PD-L1 enhancer and regulates the expression of PD-L1 transcription, and the level of PD-L1 expression was consistent with YAP transcriptional activity in A549, H1975, H1299 and SKLU-1 cell lines.

Expression of PD-L1 as an adaptive response to endogenous antitumor immunity can occur because PD-L1 is induced on most tumor cells in response to inflammatory cytokines, predominantly IFN-γ, which has been confirmed in melanoma, ovarian cancer, and part of lung cancer [42]. IFN-γ is a pro-inflammatory cytokine that regulates anti-tumor immunity and is produced mainly by activated type 1 CD4+ helper T cells (Th1), CD8+ cytotoxic T lymphocytes (CTLs), macrophages, and natural killer (NK) cells. The IFN-γ-induced PD-L signaling pathway may be involved with interferon regulatory factor-1, STAT 1/3 [43, 44]. The regulation of PD-L1 appears to be very complex and may involve multiple signaling pathways. Feng *et al*. [45] showed that lactic acid can promote PD-L1 expression and that TAZ contributes to PD-L1 regulation. We found that after YAP gene silencing, PD-L1 expression decreased significantly, but TAZ expression did not change. Immune checkpoints including PD-L1/PD-1 have are of great interest for researchers of anticancer immunotherapy. Our finding that YAP regulates PD-L1 expression in NSCLC cell lines provides a basis for exploring potential therapeutic Hippo/YAP targets. PD-L1 inhibitors combined with drugs that inhibit YAP1 activity may have a synergistic effect. Drugs that potentially regulate YAP activity include Dasatinib, Y27632, TPCA-1, AZD0532, and so on, which target YES1, GPCR-ROCK, stat3, Src/YES, and other sites [46]. Recently, the combination of the inhibitor of YAP1 activity with other drugs have shown some promising results in EGFR-mutant NSCLC mouse models [47]. Further study of the interactions between Hippo/YAP signaling pathways and immunomodulation could lead to the development of new synergistic drugs for lung cancer.

In conclusion, our study indicates that YAP and PD-L1 expression are significantly correlated in NSCLC tissues. The reason for this may be that YAP regulates the expression of PD-L1 in NSCLC at the transcriptional level. Future studies should focus on whether regulation of YAP and PD-L1 is active in a variety of tumor types and whether this should affect the choice of clinical treatment and prognosis for NSCLC in the future.

## METHODS

### Cell culture

Human NSCLC cell lines H460, H2170, SKLU-1, H1975, H1299, A549, H2030, and PC-9 were obtained from American Type Culture Collections (Manassas, VA). Cell lines were maintained in RPMI-1640 except for SKLU-1, which was maintained in EMEM. All media were supplemented with 10% heat-inactivated fetal bovine serum, penicillin (100 mg/ml). LP-9 was maintained in M199 supplemented with 15%(v/v) heat-inactivated FBS, 10 ng/ml EGF, 0.4 ug/ml hydrocortisone and penicillin (100 IU/ml). All cells were cultured at 37°C in a humid incubator with 5% CO_2_.

### RNA isolation, cDNA synthesis and quantitative real-time RT-PCR

Total RNA was extracted from cells using the RNeasy Mini kit (Qiagen, Valencia, CA). The cDNA was transcribed from 500 ng of total RNA using iScript-cDNA Synthesis Kits (Bio-Rad, Hercules, CA), according to the manufacturer’s protocol. The cDNA was used as the template for real-time PCR detection using TaqMan Technology on an Applied Biosystems 7000 sequence detection system (Applied Biosystems, Foster City, CA). Expression of PD-L1, YAP genes and endogenous control gene b-glucuronidase (GUSB) was detected by using the primer and probe sequences commercially available (Thermo Fisher Scientific, Rockford, IL) and analyzed using Relative Quantification Software (Applied Biosystems). The TaqMan Assay IDs of primer sequences for qRT-PCR were GUSB (Hs00939627 m1, Lot 1516740, 1516749 G3), PD-L1 (CD274, Hs01125301 m1, Lot 1548518, 1548524 D12) and YAP1 (Hs00902712 g1; Lot 1450809; 1450830 H6). Waltham, MA USA 02451.

### SiRNA and plasmid DNA transfection

The SMAR-Tpool siRNA targeting YAP (YAP siRNA-1) was purchased from Thermo Scientific Dharmacon (Pittsburgh, PA, USA). Non-targeting siRNA was used as control (Thermo Scientific Dharmacon). Another YAP siRNA targeting the 3′UTR end of the YAP gene (YAP siRNA-2) was purchased from Life Technologies (Grand Island, NY). The YAP plasmid DNA used to overexpress the YAP gene in the cells was purchased from Addgene (Cambridge, MA). Cells were plated in 6-well plates (for western blot) and 24-well plates (for PCR and reporter assay) for 24 hours before treatment. Cells were transfected with 4μg of YAP plasmid DNA using Lipofectamine 2000 (Invitrogen, Carlsbad, CA) transfection reagent, and 100 nmol/L of siRNA using Lipofectamine RNAiMAX (Invitrogen) according to the manufacturer’s protocol. After transfection for 48 hours, cells were harvested for further analysis.

### Luciferase reporter assay

The 8 × GTIIC-luciferase plasmid (Addgene) and Renilla luciferase pRL-TK plasmid (Promega, Madison, WI) were co-transfected into cell lines. The si-RNA transfection reagent was Lipofectamine RNAiMAX (Invitrogen). After 48 hours, cells were harvested and transferred into a 96-well plate for analysis by using the Dual-Luciferase Reporter Assay Kit (Promega). Luminescent signaling was detected on a GloMax-96 Microplate Luminometer (Promega) according to the manufacturer’s instructions.

### Western blot analysis

The following primary antibodies were used for immunoblot analysis: YAP, phospho-YAP (Ser127), Src, Lamine-b and PD-L1 from Cell Signaling, Inc. (Danvers, MA); phospho-YAP (Tyr357) from Abcam (Cambridge, MA) and Sigma-Aldrich (St. Louis, MO); TAZ and α-tubulin from Santa Cruz Biotechnology (Santa Cruz, CA). Total protein was extracted from cell lines using M-PER Mammalian Protein Extraction Reagent (Thermo Fisher Scientific, Rockford, IL), and nuclear/cytoplasm proteins extracted using a nuclear/cytoplasm extraction kit (Thermo Fisher Scientific Inc.) were supplied with Complete Protease Inhibitor Cocktails (Roche, Lewes, UK), according to the manufacturers’ protocols. The protein concentrations were measured with the Pierce BCA Protein Assay Kit (Thermo Fisher Scientific, Rockford, IL). A total of 15 μg of proteins was run on 4∼20% gradient SDS–polyacrylamide gels (Bio-Rad) and transferred to Immobilon-P nitrocellulose membranes (Millipore, Bellerica, MA). The membranes were blocked in 5% non-fat milk and then probed with the primary antibodies overnight at 4°C. The membranes were incubated with appropriate secondary antibodies, and detected by using an ECL blotting analysis system (Amersham Pharmacia Biotech, Piscataway, NJ).

### Tissue samples and immunohistochemistry

Fresh lung tumor tissues were obtained from patients who were undergoing surgical resection of a primary tumor. All human tissue samples were obtained and analyzed in accordance with procedures approved by the institutional review board of the University of California, San Francisco (IRB H8714–22 942–01). The tissue microarray sections were immunostained as previously described [48]. The following scoring system was used: −, no stain; +, weak staining (10% or above stained cellularity considered as positive); ++, moderate staining (30% or above stained cellularity considered as positive); +++, strong staining (50% or above stained cellularity considered as positive). All scoring was done under low power objective lens (10×) with a Zeiss Axioscop 2 microscope (Carl Zeiss Inc, Germany). Images were taken under 10× or 20× objective lens.

### Immunofluorescence

Cells were seeded on a Chamber SlideTM (Thermo Fisher Scientific) at 50% confluence and fixed with 100% methanol following their respective treatments. After permeabilization using Triton-X100, slides were subsequently blocked for 1 h at room temperature with Dulbecco’s phosphate-buffered saline (PBS) containing 5% bovine serum albumin (BSA) and incubated with primary antibody overnight at 4°C. Antibodies were diluted in PBS containing 5% BSA. Primary antibodies of YAP and phospho-YAP (Ser127) were purchased from Cell Signaling, Inc. (Danvers, MA). After being washed, the slides were incubated with the corresponding secondary antibodies in the dark for 1 h at room temperature, washed again, and mounted using ProLong Antifade with DAPI to visualize the nuclei. The slides were analyzed using a fluorescent confocal microscope equipped with an ultraviolet laser (Carl Zeiss Inc, Germany).

### ChIP assay

Fragmented chromatin from SKLU-1 and H1299 cells was incubated with anti-IgG (negative control), anti-YAP, and anti-POL-II (positive control). Fragmented chromatin from SKLU-1, H1299, H1975 and A549 cells was incubated with anti-YAP. Recruited DNA was subjected to PCR using the primers for distal enhancer regions of PD-L1, and PCR products were electrophoresed in agarose gel. The ChIP assay was conducted using the Chromatin Immunoprecipitation (ChIP) Assay Kit (Millipore Corporation). Monoclonal antibodies for YAP (Cell Signaling Technology) and control rabbit antibody for IgG (Cell Signaling Technology) were used for ChIP. Two pairs’ primer used for RT-PCR to amplify the PD-L1 gene, one pair primers were 5′-TCGGTCTGTGAAGGACTGC-3′ and 5′-ACCGTTGAGGAATGGATGAA-3′ resulting in a product size of 203 bp, and the other were 5′-CCACCACCATTATCTAATTCCA-3′ and 5′-AAGGAGCCAGACACAAAAGG-3′ resulting in a product size of 210 bp.

### Statistical analysis

Data are expressed as mean ± standard deviation (SD) from independent experiments. All statistical analyses were performed using the SPSS 23.0 for Windows software system (SPSS Inc, Chicago, IL). One-way ANOVA followed by Scheffe multiple comparisons were used to compare the differences among multiple groups. Statistical significance of differences between groups was determined by Student’s *t*-test. The relationship between YAP and PD-L1 was analyzed with Spearman product correlation. For all analyses, statistical significance was defined as *P* < 0.05 (^*^P < 0.05, ^**^P ≤ 0.01, ^***^P ≤ 0.001), based on two-tailed tests.

## SUPPLEMENTARY TABLES AND FIGURE




