# Supplementary material for: YAP regulates PD-L1 expression in human NSCLC cells
Source: Oncotarget. 2017 Dec 9;8(70):114576–87. doi: 10.18632/oncotarget.23051 (PMC5777715; doi:10.18632/oncotarget.23051)
Supplement: Supplementary file 1 [file oncotarget-08-114576-s001.pdf]

YAP regulates PD-L1 expression in human NSCLC cells

SUPPLEMENTARY MATERIALS

Supplementary Table 1: Immunohistochemistry findings and basic information from human non-small cell lung cancer samples. See Supplementary_Table_1

Supplementary Table 2: AP IHC comparison in patients with different clinical characteristics

Clinical characteristics	YAP		X^2	*P* value
	Positive (++/+++)	Negative (–/+)		
**TNM stage***			1.706	0.653
I	30	18		
II	17	10		
III	12	4		
IV	2	0		
**Histopathology**			0.027	0.869
Squamous	33	21		
Non-squamous	55	33		

*In 142 patients, there were no available TNM in 45 cases, 97 cases can be analyzed.

Supplementary Table 3: PD-L1 IHC comparison in patients with different clinical characteristics

Clinical characteristics	PD-L1		X^2	*P* value
	Positive (++/+++)	Negative (–/+)		
**TNM stage***			0.302	0.96
I	20	32		
II	10	17		
III	7	9		
IV	1	1		
**Histopathology**			2.935	0.087
Squamous	15	39		
Non-squamous	37	51		

*In 142 patients, there were no available TNM in 45 cases, 97 cases can be analyzed.

Supplementary Table 4: Statistical analysis of YAP mRNA level percentage by control

Cell line	mean ± SD (percentage by control)	Statistical analysis
LP-9	1.00 ± 0.04	@@, §§§, $$$, &&&, ^^^, ¤¤¤, ###, +++
H2030	3.59 ± 0.68	$$, &&&, ^^^, ¤¤¤, ###, +++
A549	4.14 ± 0.85	$$, &&&, ^^^, ¤¤¤, ###, +++
H2170	6.27 ± 0.78	¤¤¤, ###, +++
H1975	6.89 ± 1.34	¤¤¤, ###, +++
PC9	7.06 ± 0.45	¤¤¤, ###, +++
H460	10.16 ± 1.27	###, +++
SKLU-1	19.57 ± 0.46	
H1299	20.31 ± 1.27	

VS LP-9:	*$P < 0.05$	**$P < 0.01$	***$P < 0.001$
VS H2030:	@$P < 0.05$	@@$P < 0.01$	@@@$P < 0.001$
VS A549:	§$P < 0.05$	§§$P < 0.01$	§§§$P < 0.001$
VS H2170:	$$P < 0.05$	$$$P < 0.01$	$$$$P < 0.001$
VS H1975:	&$P < 0.05$	&&$P < 0.01$	&&&$P < 0.001$
VS PC9:	^$P < 0.05$	^^$P < 0.01$	^^^$P < 0.001$
VS H460:	¤$P < 0.05$	¤¤$P < 0.01$	¤¤¤$P < 0.001$
VS SKLU-1:	# $P < 0.05$	## $P < 0.01$	### $P < 0.001$
VS H1299:	+$P < 0.05$	++$P < 0.01$	+++$P < 0.001$

Supplementary Table 5: Statistical analysis of PD-L1 mRNA level percentage by control

Cell line	mean ± SD (percentage by control)	Statistical analysis
LP-9	1.00 ± 0.19	¤¤¤, ##, ++
H2030	0.44 ± 0.13	$$, ¤¤¤, ##, +++
A549	0.61 ± 0.09	$, ¤¤, ##, +++
H2170	1.86 ± 0.17	&, ^, ¤¤¤, #, +
H1975	0.79 ± 0.01	^^, ¤¤, #, ++
PC9	0.47 ± 0.02	¤¤, #, ++
H460	8.39 ± 0.35	###, ++
SKLU-1	3.21 ± 0.22	
H1299	3.05 ± 0.11	

VS LP-9:	$^{*}P < 0.05$	$^{**}P < 0.01$	$^{***}P < 0.001$
VS H2030:	@$P < 0.05$	@@$P < 0.01$	@@@$P < 0.001$
VS A549:	§$P < 0.05$	§§$P < 0.01$	§§§$P < 0.001$
VS H2170:	$$P < 0.05	$$$P < 0.01	$$$$P < 0.001
VS H1975:	&$P < 0.05$	&&$P < 0.01$	&&&$P < 0.001$
VS PC9:	^$P < 0.05$	^^$P < 0.01$	^^^$P < 0.001$
VS H460:	¤$P < 0.05$	¤¤$P < 0.01$	¤¤¤$P < 0.001$
VS SKLU-1:	#$P < 0.05$	##$P < 0.01$	###$P < 0.001$
VS H1299:	+$P < 0.05$	++$P < 0.01$	+++$P < 0.001$

Supplemental Table 6: Statistical analysis of GTIIC reporter activity (fold)

Cell line	mean ± SD (percentage by control)	Statistical analysis
LP-9	0.90 ± 0.01	@@@, §§§, $$, &&&, ^, ¤¤¤, ##, +++
H2030	2.38 ± 0.07	§§§, $, ^^^, ¤¤¤, ##, +++
A549	0.71 ± 0.03	$$, &&&, ¤¤, ^, ¤¤¤, ###, +++
H2170	9.54 ± 1.23	&, ^^, #, +
H1975	2.59 ± 0.06	^^^, ¤¤¤, ##, +++
PC9	0.84 ± 0.00	¤¤¤, ###, +++
H460	10.80 ± 0.19	###, +++
SKLU-1	4.05 ± 0.19	
H1299	3.84 ± 0.14	

VS LP-9:	$^{*}P < 0.05$	$^{**}P < 0.01$	$^{***}P < 0.001$
VS H2030:	@$P < 0.05$	@@$P < 0.01$	@@@$P < 0.001$
VS A549:	§$P < 0.05$	§§$P < 0.01$	§§§$P < 0.001$
VS H2170:	$$P < 0.05	$$$P < 0.01	$$$$P < 0.001
VS H1975:	&$P < 0.05$	&&$P < 0.01$	&&&$P < 0.001$
VS PC9:	^$P < 0.05$	^^$P < 0.01$	^^^$P < 0.001$
VS H460:	¤$P < 0.05$	¤¤$P < 0.01$	¤¤¤$P < 0.001$
VS SKLU-1:	#$P < 0.05$	##$P < 0.01$	###$P < 0.001$
VS H1299:	+$P < 0.05$	++$P < 0.01$	+++$P < 0.001$

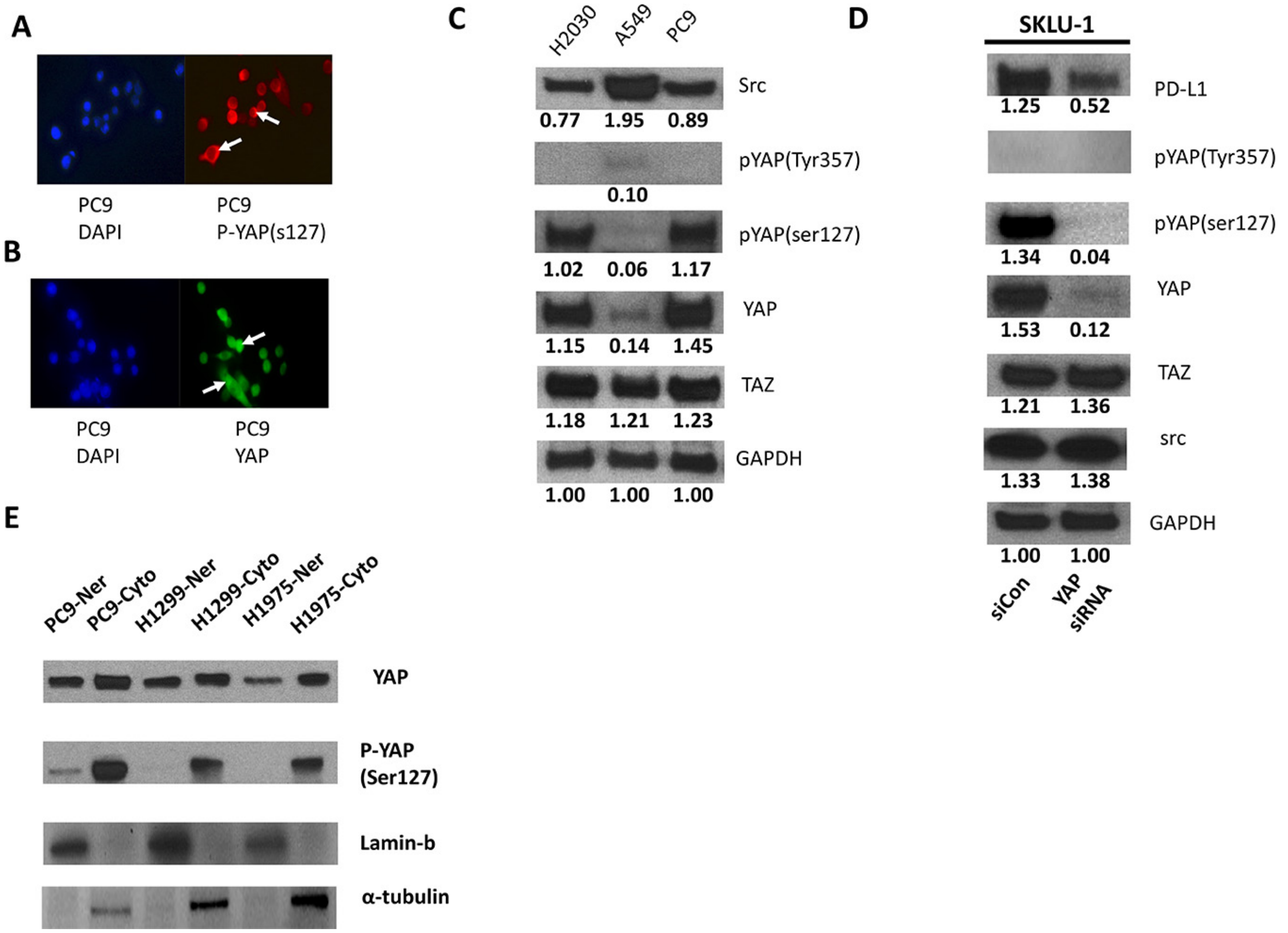

Supplementary Figure 1: pYAP and YAP expression in NSCLC cell lines. (**A**–**B**) Representative image showing expression of pYAP (ser127) protein (A) and YAP protein (B) in PC9 cell line analyzed by immunofluorescence. YAP was stained in both nucleus and cytoplasm, whereas pYAP (ser127) was found in the cytoplasm. (**C**) Western blot was used to detect levels of YAP, pYAP (ser127), pYAP (Try357), Src and TAZ in H2030, A549 and PC9 cell lines. GAPDH was detected as a loading control. Band intensity was analyzed with ImageJ software and normalized with the intensity of the GAPDH band. (**D**) Western blot analysis of PD-L1, YAP, pYAP (ser127), pYAP (Try357), Src and TAZ in SKLU-1 cell line transfected with YAP siRNA or control siRNA. GAPDH was detected as a loading control. Band intensity was analyzed with ImageJ software and normalized with the intensity of the GAPDH band. (**E**) Western Blot analysis YAP, pYAP (ser127) in nucleus and cytoplasm of PC9, H1975 and H1299 cell lines. Lamin-b and α-tubulin were detected as a loading control.
