# Supplementary material for: YAP regulates PD-L1 expression in human NSCLC cells
Source: Oncotarget. 2017 Dec 9;8(70):114576–87. doi: 10.18632/oncotarget.23051 (PMC5777715; doi:10.18632/oncotarget.23051)
Supplement: Supplementary file 2 [file oncotarget-08-114576-s002.docx]

| Sample  **Supplemental Table S1:**  **Immunohistochemistry findings and basic information from human non-small cell lung cancer samples**  NO. | IHC of  anti-YAP | IHC of anti-PD-L1 | Histopathology | TNM  stage | IHC of EGFR |
| --- | --- | --- | --- | --- | --- |
| T01 | ++ | +++ | ADE | II | - |
| T02 | ++ | + | ADE | I | - |
| T03 | ++ | ++ | ADE+  SQU | I | - |
| T04 | ++ | +++ | ADE | X | + |
| T05 | ++ | ++ | ADE | I | + |
| T06 | ++ | +++ | ADE | X | - |
| T07 | ++ | ++ | ADE | IIB | - |
| T08 | ++ | +++ | ADE | I | ++ |
| T09 | - | - | ADE | X | - |
| T10 | - | - | ADE | IIIA | - |
| T11 | - | - | ADE | X | - |
| T12 | ++ | ++ | ADE | IIIA | + |
| T13 | - | - | ADE | II | - |
| T14 | + | + | ADE | X | - |
| T15 | +++ | +++ | ADE | IIIB | - |
| T16 | ++ | ++ | ADE | IIIA | - |
| T17 | ++ | ++ | ADE | X | +++ |
| T18 | + | + | ADE | IIIB | - |
| T19 | - | - | ADE | I | - |
| T20 | - | ++ | ADE | II | - |
| T21 | ++ | +++ | ADE | I | - |
| T22 | ++ | ++ | ADE | X | - |
| T23 | ++ | ++ | ADE | I | ++ |
| Sample  NO. | IHC of  anti-YAP | IHC of anti-PD-L1 | Histopathology | TNM  stage | IHC of EGFR |
| T24 | ++ | + | ADE | I | - |
| T25 | ++ | + | ADE | II | - |
| T26 | + | - | ADE | I | +++ |
| T27 | ++ | +++ | ADE | I | - |
| T28 | +++ | + | ADE | IIIA | - |
| T29 | ++ | + | ADE | II | - |
| T30 | + | + | ADE | I | - |
| T31 | +++ | - | ADE | X | - |
| T32 | +++ | - | ADE | IV | +++ |
| T33 | ++ | + | ADE | II | - |
| T34 | - | - | ADE | X | - |
| T35 | - | + | ADE | III | - |
| T36 | ++ | + | ADE | X | ++ |
| T37 | ++ | + | ADE | IIIA | - |
| T38 | - | - | ADE | I | - |
| T39 | ++ | - | ADE | X | - |
| T40 | ++ | + | ADE | X | - |
| T41 | ++ | ++ | ADE | II | - |
| T42 | + | ++ | ADE | I | - |
| T43 | ++ | + | ADE | X | ++ |
| T44 | +++ | ++ | ADE | X | ++ |
| T45 | - | + | ADE | I | - |
| T46 | - | + | ADE | I | - |
|  |  |  |  |  | - |
| Sample  NO. | IHC of  anti-YAP | IHC of anti-PD-L1 | Histopathology | TNM  stage | IHC of EGFR |
| T47 | + | + | ADE | IA | - |
| T48 | ++ | + | ADE | I | - |
| T49 | ++ | + | ADE | IIA | + |
| T50 | + | + | ADE | I | - |
| T51 | +++ | +++ | ADE | I | - |
| T52 | ++ | + | ADE | X | + |
| T53 | - | + | ADE | X | - |
| T54 | + | + | ADE | X | +++ |
| T55 | +++ | + | ADE | I | - |
| T56 | + | + | ADE | I | - |
| T57 | - | ++ | ADE | IIIA | ++ |
| T58 | - | - | ADE | II | - |
| T59 | +++ | +++ | ADE | I | - |
| T60 | + | + | ADE | II | - |
| T61 | +++ | +++ | ADE | I | - |
| T62 | ++ | +++ | ADE | I | - |
| T63 | + | ++ | ADE | X | - |
| T64 | +++ | +++ | ADE | II | - |
| T65 | +++ | + | ADE | II | ++ |
| T66 | ++ | + | ADE | IIB | + |
| T67 | ++ | ++ | ADE | II | - |
| T68 | - | - | ADE | I | - |
| T69 | ++ | +++ | ADE | X | + |
|  |  |  |  |  |  |
| Sample  NO. | IHC of  anti-YAP | IHC of anti-PD-L1 | Histopathology | TNM  stage | IHC of EGFR |
| T70 | ++ | +++ | ADE | I | - |
| T71 | ++ | +++ | ADE | X | - |
| T72 | ++ | ++ | ADE | II | - |
| T73 | ++ | + | ADE | X | - |
| T74 | ++ | ++ | ADE | I | - |
| T75 | - | - | ADE | X | - |
| T76 | +++ | +++ | ADE | IIA | - |
| T77 | - | - | ADE | X | - |
| T78 | + | - | ADE | X | - |
| T79 | + | + | ADE | I | - |
| T80 | +++ | ++ | ADE | X | ++ |
| T81 | + | - | ADE | X | - |
| T82 | +++ | +++ | NEUROENDOCRINE CARCINOMA | III | X |
| T83 | +++ | ++ | LARGE CELL CARCINOMA | X | X |
| T84 | +++ | ++ | SQU | IV | X |
| T85 | +++ | +++ | SQU+LARGE CELL | I | X |
| T86 | + | + | SQU+LARGE CELL | X | X |
| T87 | ++ | + | SQU+LARGE CELL | I | X |
| Sample  NO. | IHC of  anti-YAP | IHC of anti-PD-L1 | Histopathology | TNM  stage | IHC of EGFR |
| T88 | +++ | +++ | SQU | II | X |
| T89 | + | - | SQU | X | X |
| T90 | +++ | +++ | SQU | III | X |
| T91 | + | ++ | SQU | X | X |
| T92 | +++ | ++ | SQU | X | X |
| T93 | ++ | + | SQU | IIIB | X |
| T94 | + | + | SQU | II | X |
| T95 | + | + | SQU | II | X |
| T96 | - | - | SQU | I | X |
| T97 | - | - | SQU | X | X |
| T98 | - | - | SQU | X | X |
| T99 | ++ | + | SQU | I | X |
| T100 | ++ | - | SQU | X | X |
| T101 | ++ | + | SQU | I | X |
| T102 | ++ | + | SQU | IB | X |
| T103 | ++ | ++ | SQU | I | X |
| T104 | ++ | + | SQU | IIIA | X |
| T105 | ++ | + | SQU | I | X |
| T106 | ++ | + | SQU | I | X |
| T107 | ++ | + | SQU | I | X |
| T108 | ++ | + | SQU | II | X |
| T109 | + | + | SQU | I | X |
| T110 | + | + | SQU | II | X |
|  |  |  |  |  |  |
| Sample  NO. | IHC of  anti-YAP | IHC of anti-PD-L1 | Histopathology | TNM  stage | IHC of EGFR |
| T111 | + | ++ | SQU | IB | X |
| T112 | +++ | + | SQU | X | X |
| T113 | +++ | ++ | SQU | X | X |
| T114 | +++ | ++ | SQU | I | X |
| T115 | +++ | ++ | SQU | I | X |
| T116 | +++ | + | SQU | I | X |
| T117 | +++ | ++ | SQU | IIIB | X |
| T118 | ++ | - | SQU | II | X |
| T119 | ++ | - | SQU | I | X |
| T120 | ++ | - | SQU | IIIA | X |
| T121 | ++ | + | SQU | I | X |
| T122 | ++ | + | SQU | I | X |
| T123 | ++ | ++ | SQU | I | X |
| T124 | ++ | + | SQU | I | X |
| T125 | ++ | - | SQU | IIA | X |
| T126 | ++ | + | SQU | IB | X |
| T127 | - | ++ | SQU | I | X |
| T128 | - | - | SQU | II | X |
| T129 | - | + | SQU | I | X |
| T130 | - | - | SQU | X | X |
| T131 | + | ++ | SQU | II | X |
| T132 | - | ++ | SQU | X | X |
| T133 | - | - | SQU | IA | X |
|  |  |  |  |  |  |

| Sample  NO. | IHC of  anti-YAP | IHC of anti-PD-L1 | Histopathology | TNM  stage | IHC of EGFR |
| --- | --- | --- | --- | --- | --- |
| T134 | + | + | SQU | X | X |
| T135 | ++ | + | SQU | X | X |
| T136 | ++ | + | SQU | X | X |
| T137 | ++ | + | SQU | IIIA | X |
| T138 | - | - | ADE | II | X |
| T139 | ++ | + | ADE | X | X |
| T140 | + | + | SQU | X | X |
| T141 | + | + | SQU | X | X |
| T142 | + | + | SQU | X | X |
| N1 | - | + | N | X | X |
| N2 | + | + | N | X | X |
| N3 | - | + | N | X | X |
| Sample  NO. | IHC of  anti-YAP | IHC of anti-PD-L1 | Histopathology | TNM  stage | IHC of EGFR |
| N4 | - | - | N | X | X |
| N5 | - | - | N | X | X |
| N6 | - | + | N | X | X |
| N7 | - | - | N | X | X |
| N8 | - | - | N | X | X |
| N9 | - | + | N | X | X |
| N10 | + | - | N | X | X |
| N11 | - | - | N | X | X |
| N12 | - | - | N | X | X |
| N13 | - | + | N | X | X |
| N14 | - | - | N | X | X |
| N15 | - | - | N | X | X |

**N=normal tissue; T=tumor tissue; IHC=immunohistochemistry;**

**- =no stain; + =weak stain; ++ =moderate stain; +++ =strong stain**

**X= not available; ADE= adenocarcinoma; SQU= squamous cell carcinoma**
